# Supplementary figures and images for: Individualized spatial network predictions using Siamese convolutional neural networks: A resting-state fMRI study of over 11,000 unaffected individuals
Source: PLoS One. 2022 Jan 21;17(1):e0249502. doi: 10.1371/journal.pone.0249502 (PMC8782493; doi:10.1371/journal.pone.0249502)

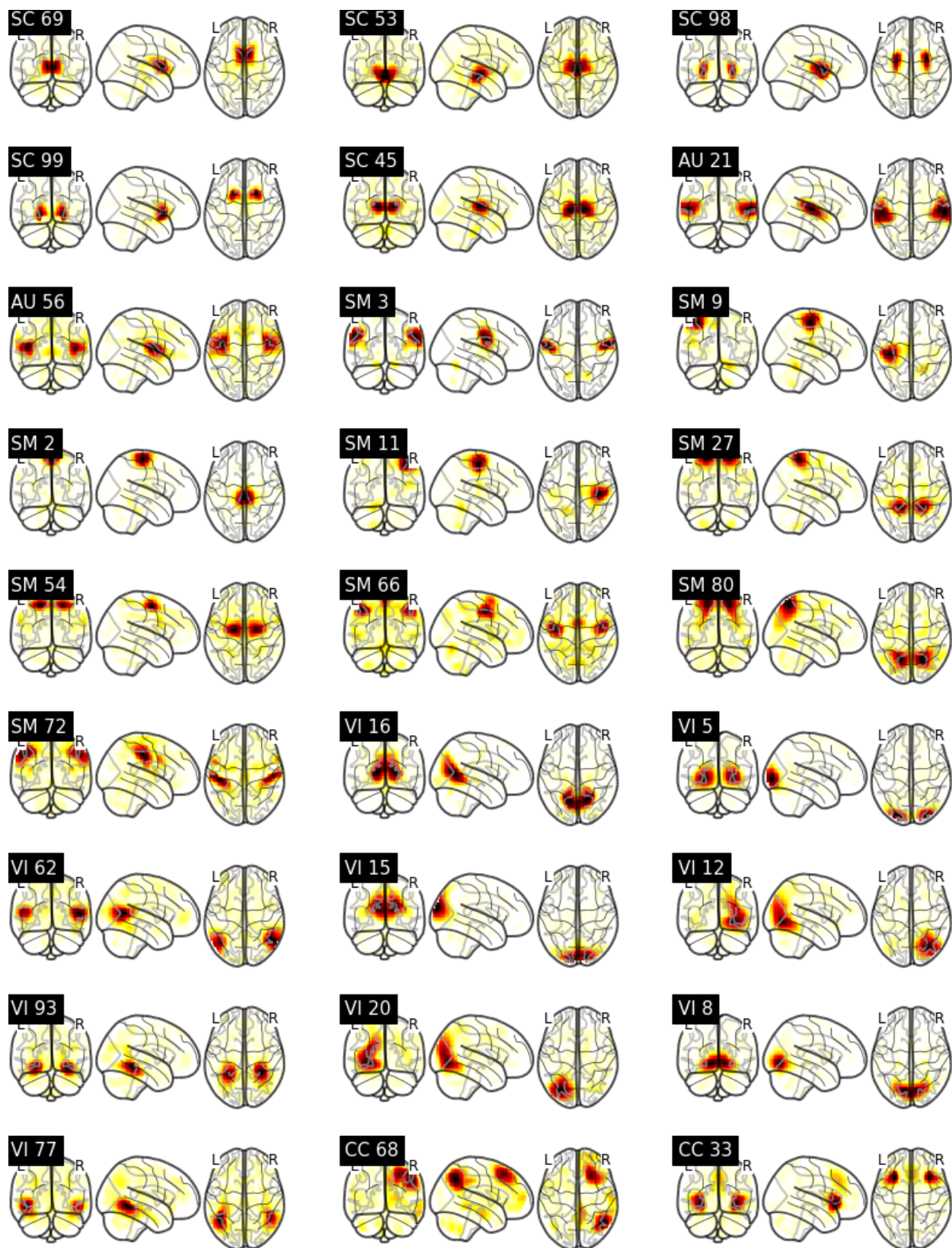

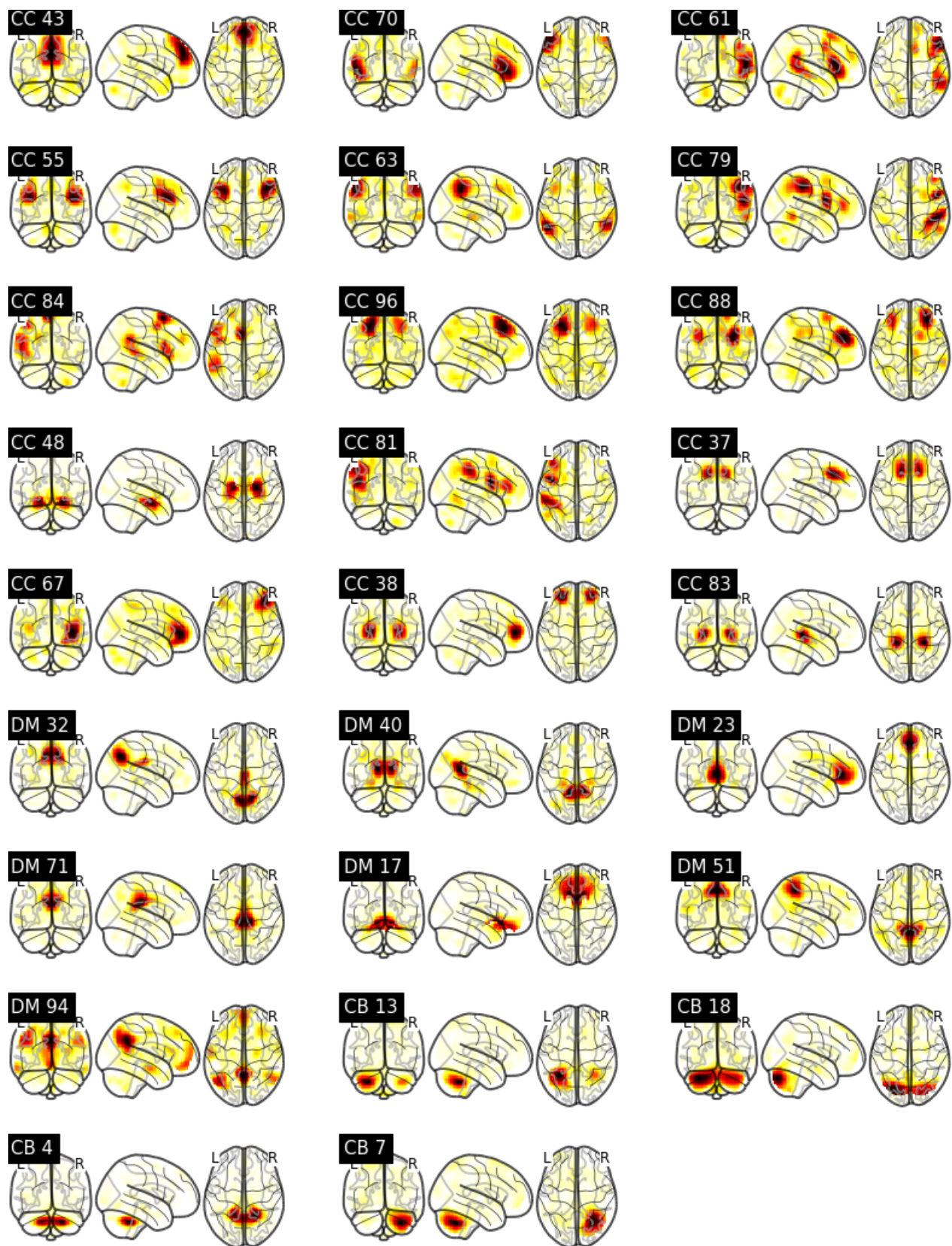

Supplement: S1 Fig — (PDF) [file pone.0249502.s001.pdf]

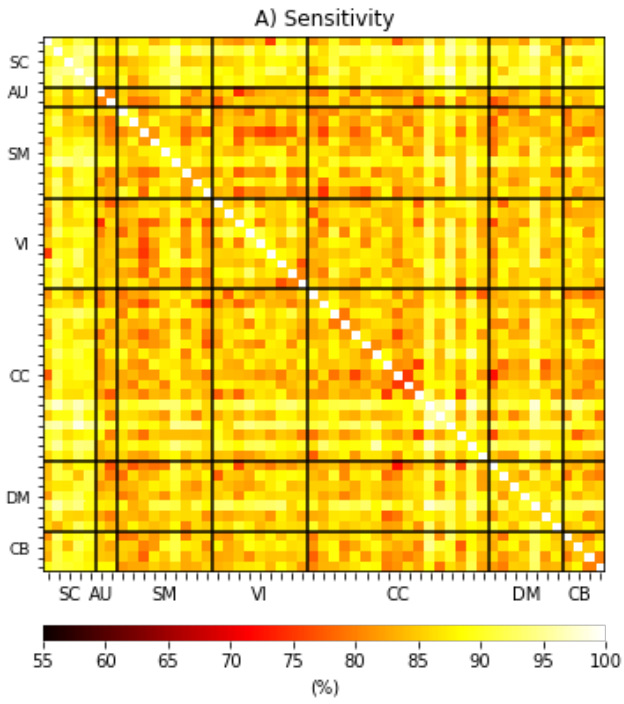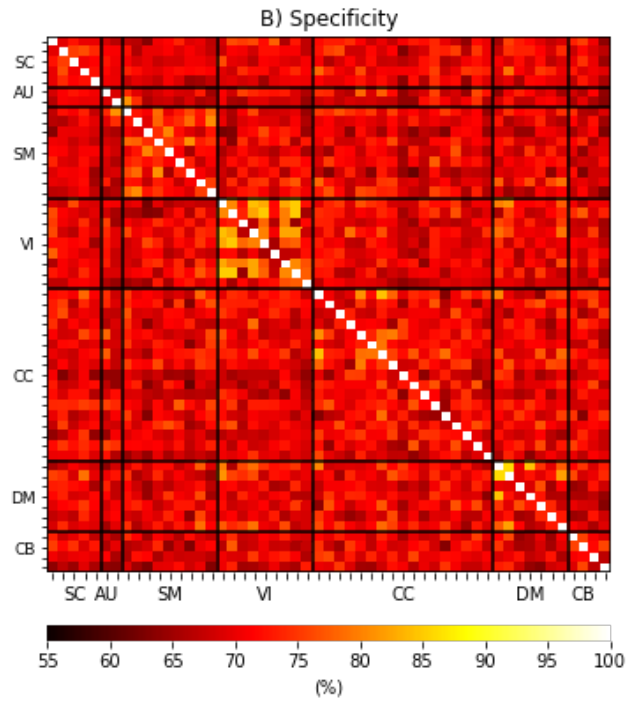

Supplement: S3 Fig — S3A and S3B Fig depict the heatmap of sensitivity and specificity, respectively, expressed as percentages. Comparing the two heatmaps reveals an interesting observation that certain domains (here, the subcortical domain) appear to contribute more significantly when classifying ‘same subjects,’ while a different set of networks (especially VI-VI and SM-SM network pairs in S3B Fig) contribute the most toward classification of ‘different subjects’. (PDF) [file pone.0249502.s003.pdf]

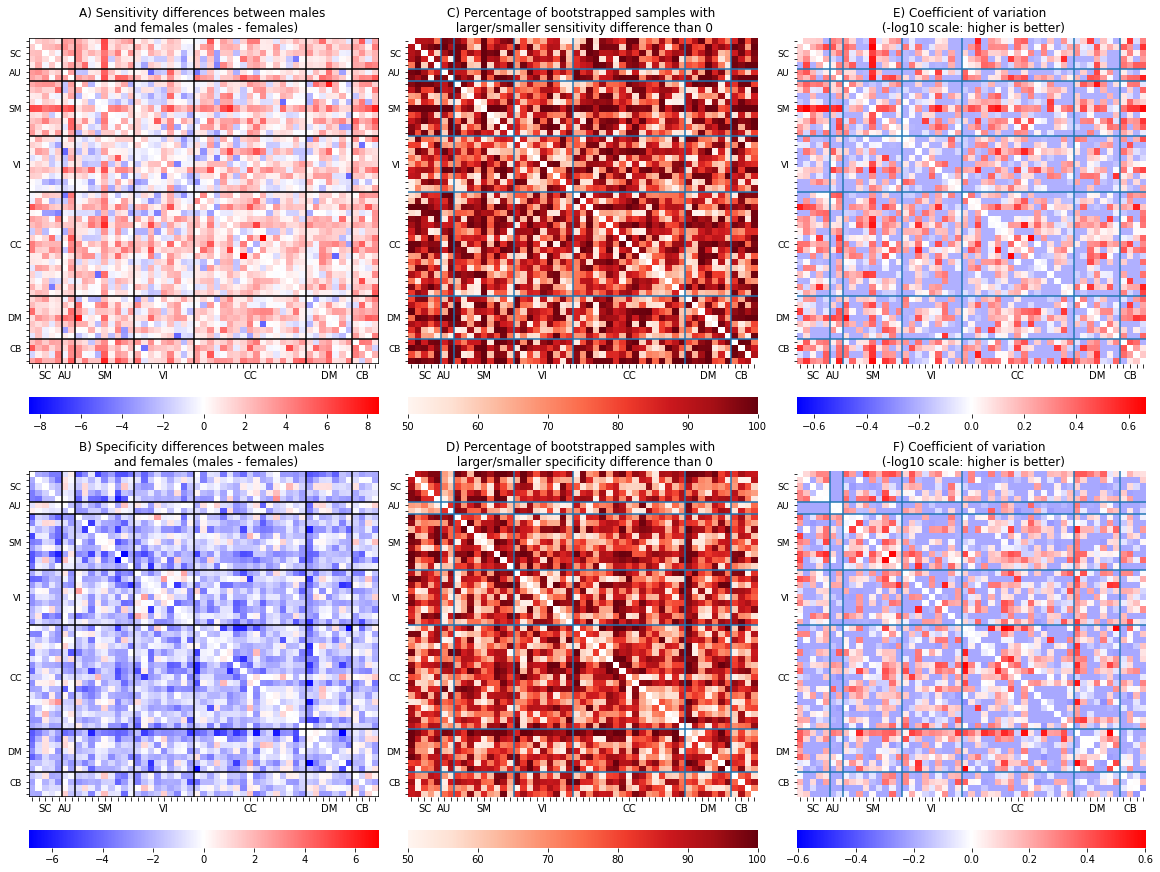

Supplement: S4 Fig — Fig 4A shows the sensitivity differences computed from subtracting the sensitivity of females from that of males. According to the figure, network pair samples coming from male subjects perform better (i.e., they attain higher sensitivity) than samples coming from female subjects in most cases of same-subject prediction. Fig 4B depicts the specificity differences computed from subtracting the different-sex specificities from same-sex specificities. This figure shows that when subjects have different sex, the model performs better (higher specificity) in different-subject prediction than when subjects are from the same sex. Note that sensitivity and specificity are expressed as percentages. (C-D) Percentage of bootstrapped differences larger/smaller than 0: (C) corresponds to Fig A and (D) corresponds to Fig B. Bootstrapped sensitivity and specificity differences were evaluated 1,000,000 times (with replacement) to assess how likely it is to observe a non-zero value in sensitivity/specificity difference per network pair, in the direction of the original difference shown in panels (A) and (B). Large percentages indicate the directionality of the observed difference replicates reliably. Note that we report the number of times values are greater or smaller than zero (whichever is bigger). Thus, percentages closer to 50% are indicative of unreliable (chance) replication of the original result. (E-F) Coefficient of variation. Fig 4E and 4F report the ratio of the standard deviation to the absolute (unsigned) mean of bootstrapped samples for sensitivity and specificity differences, respectively. For bootstrapped mean differences whose absolute value are below 1, if their standard deviation exceeds the mean by 1.6 times or more, we simply report -10log10(1.6) = -0.2. As illustrated, differences with larger absolute value have lower bootstrapped variability, further supporting their reliability. (PNG) [file pone.0249502.s004.png]

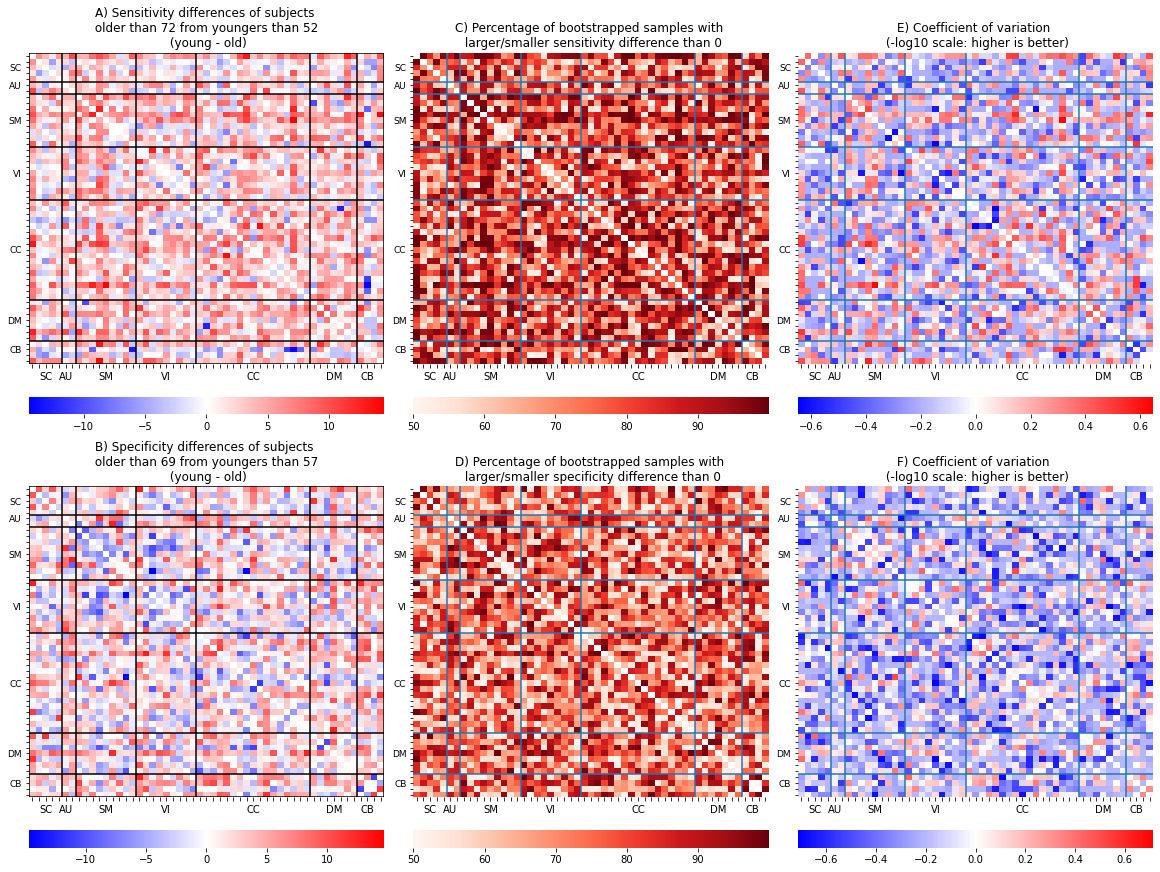

Supplement: S5 Fig — Fig 5A and 5B show the sensitivity and specificity score differences obtained by deducting the scores of old subjects from those of young subjects. According to the results, for most network pairs the sensitivity and specificity scores are higher in young subjects than in old subjects. Note that sensitivity and specificity are expressed as percentages. (C-D) Percentage of bootstrapped differences larger/smaller than 0: (C) corresponds to Fig A and (D) corresponds to Fig B. Bootstrapped sensitivity and specificity differences were evaluated 1,000,000 times (with replacement) to assess how likely it is to observe a non-zero value in sensitivity/specificity difference per network pair, in the direction of the original difference shown in panels (A) and (B). Large percentages indicate the directionality of the observed difference replicates reliably. Note that we report the number of times values are greater or smaller than zero (whichever is bigger). Thus, percentages closer to 50% are indicative of unreliable (chance) replication of the original result. (E-F) Coefficient of variation. Fig 5E and 5F report the ratio of the standard deviation to the absolute (unsigned) mean of bootstrapped samples for sensitivity and specificity differences, respectively. For bootstrapped mean differences whose absolute value are below 1, if their standard deviation exceeds the mean by 1.6 times or more, we simply report -10log10(1.6) = -0.2. As illustrated, in (E) sensitivity differences with larger absolute value have lower bootstrapped variability, further supporting their reliability, while in (F) large negative specificity differences (old > young) appear to have lower bootstrapped variability than equally-large positive specificity differences. (PNG) [file pone.0249502.s005.png]
