# Supplementary material for: Individualized spatial network predictions using Siamese convolutional neural networks: A resting-state fMRI study of over 11,000 unaffected individuals
Source: PLoS One. 2022 Jan 21;17(1):e0249502. doi: 10.1371/journal.pone.0249502 (PMC8782493; doi:10.1371/journal.pone.0249502)
Supplement: S2 Fig — Each entry in the heatmap indicates the accuracy of a network pair on the entire test set. The heatmap is grouped according to their functional domains, i.e., SC, AU, SM, VI, CC, DM, and CB which contain 5, 2, 9, 9, 17, 7, and 4 networks, respectively. The results suggest that different networks have different amount of discriminative information for characterizing subjects. For example, the subcortical, and visual networks appear to be more predictive (i.e., have more discriminative information) than auditory networks when paired with a broad range of networks across all domains. (PDF) [file pone.0249502.s002.pdf]

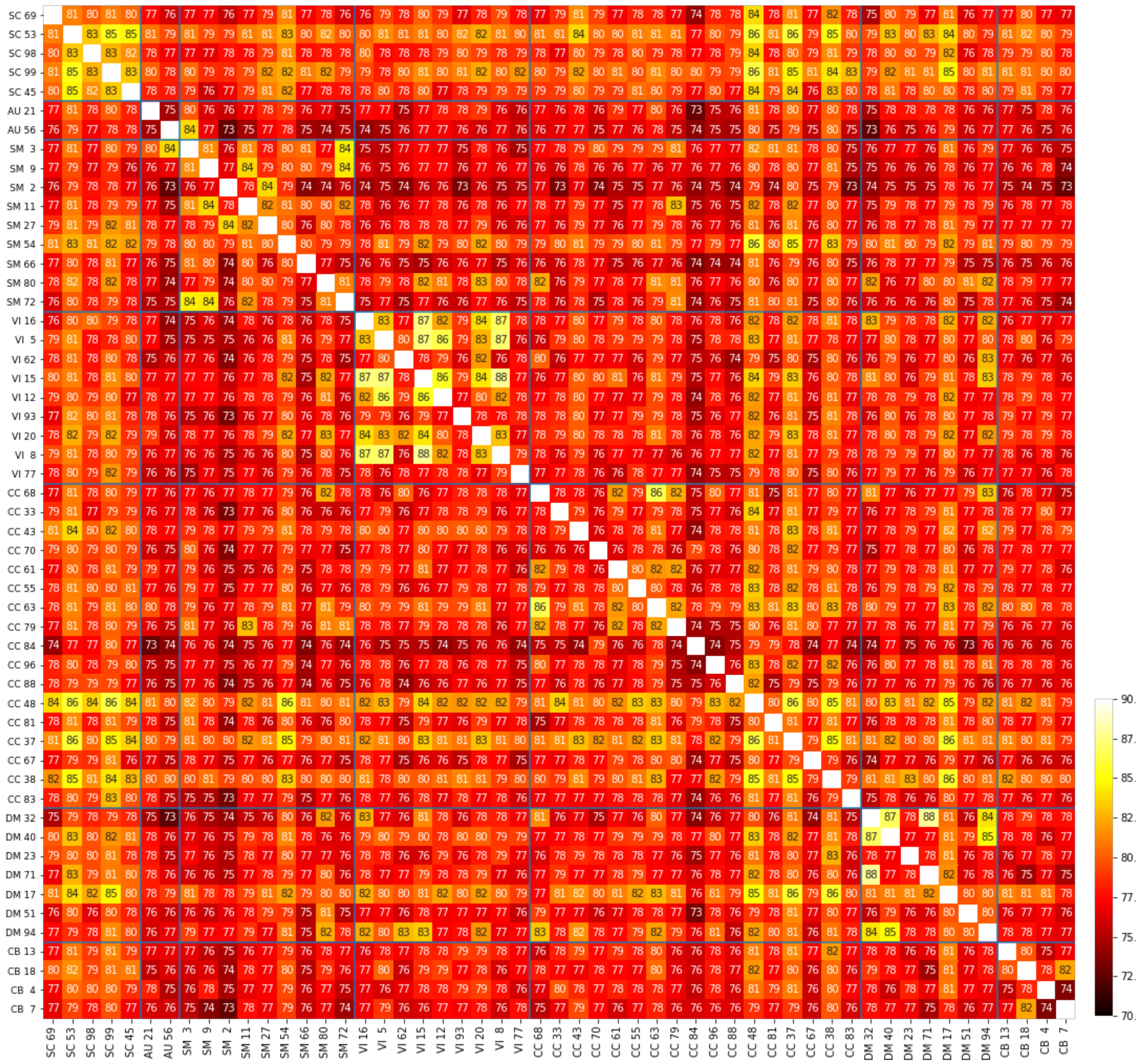
SC 69
SC 53
SC 98
SC 99
SC 45
AU 21
AU 56
SM 3
SM 9
SM 2
SM 11
SM 27
SM 54
SM 66
SM 80
SM 72
VI 16
VI 5
VI 62
VI 15
VI 12
VI 93
VI 20
VI 8
VI 77
CC 68
CC 33
CC 43
CC 70
CC 61
CC 55
CC 63
CC 79
CC 84
CC 96
CC 88
CC 48
CC 81
CC 37
CC 67
CC 38
CC 83
DM 32
DM 40
DM 23
DM 71
DM 17
DM 51
DM 94
CB 13
CB 18
CB 4
CB 7
90.0
87.5
85.0
82.5
80.0
77.5
75.0
72.5
70.0
