# Supplementary material for: Individualized spatial network predictions using Siamese convolutional neural networks: A resting-state fMRI study of over 11,000 unaffected individuals
Source: PLoS One. 2022 Jan 21;17(1):e0249502. doi: 10.1371/journal.pone.0249502 (PMC8782493; doi:10.1371/journal.pone.0249502)
Supplement: S6 Fig — Fig A shows that the model’s sensitivity in males is higher than females in all brain domains, especially in CB, where 87% of network pairs have higher sensitivity in males than females. (B) Different-sex brains are more variable than same-sex brains. Fig B shows that most network pairs in the different-sex cohort have higher specificity than those in the same-sex cohort within domains, especially in DM, SM, VI, and SC. (C-D) Younger brains are more variable than elders’. According to Fig C and D, sensitivity and specificity in young brains are higher than in old brains. (PDF) [file pone.0249502.s006.pdf]

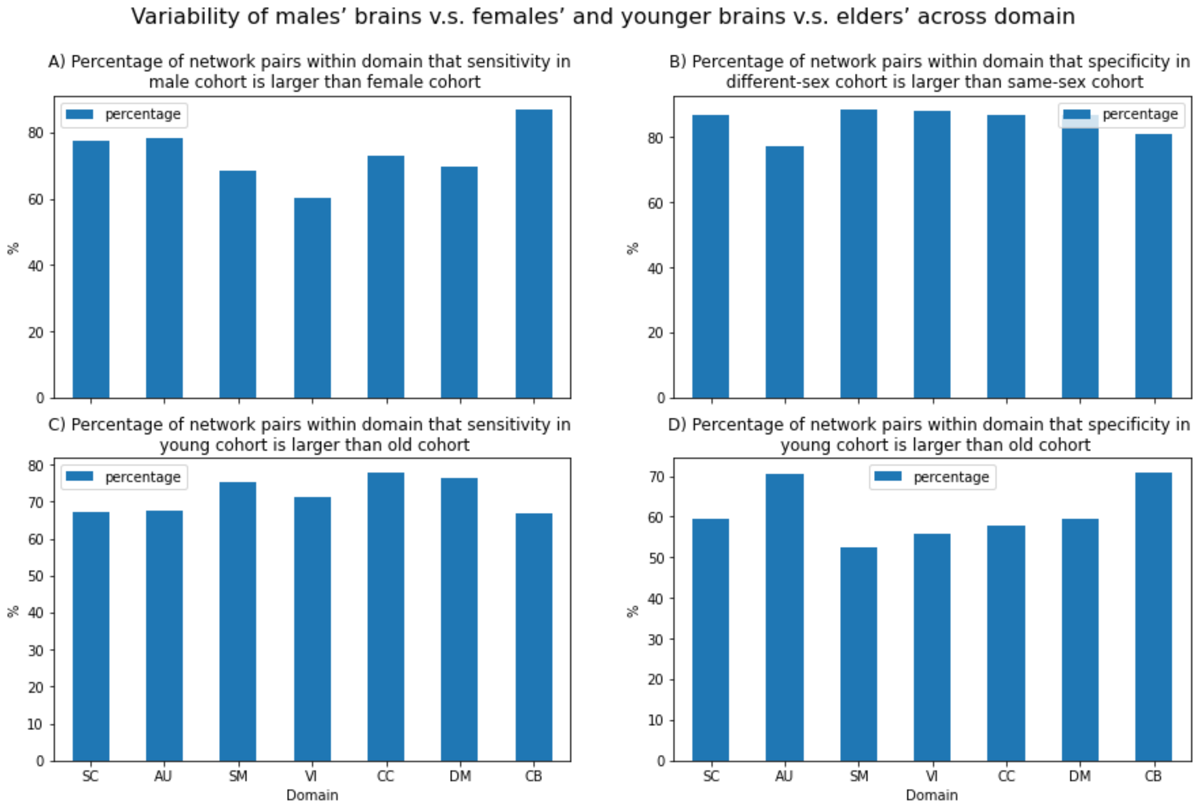

Variability of males' brains v.s. females' and younger brains v.s. elders' across domain
A) Percentage of network pairs within domain that sensitivity in male cohort is larger than female cohort
B) Percentage of network pairs within domain that specificity in different-sex cohort is larger than same-sex cohort
C) Percentage of network pairs within domain that sensitivity in young cohort is larger than old cohort
D) Percentage of network pairs within domain that specificity in young cohort is larger than old cohort
percentage
%
Domain
SC
AU
SM
VI
CC
DM
CB
