# Supplementary material for: Individualized spatial network predictions using Siamese convolutional neural networks: A resting-state fMRI study of over 11,000 unaffected individuals
Source: PLoS One. 2022 Jan 21;17(1):e0249502. doi: 10.1371/journal.pone.0249502 (PMC8782493; doi:10.1371/journal.pone.0249502)
Supplement: S1 Table — (DOCX) [file pone.0249502.s007.docx]

| **ID** | **Region** |
| --- | --- |
| SC 68 | Caudate |
| SC 53 | Subthalamus/hypothalamus |
| SC 98 | Putamen |
| SC 99 | Caudate |
| SC 45 | Thalamus |
| AU 21 | Superior temporal gyrus |
| AU 56 | Middle temporal gyrus |
| SM 3 | Postcentral gyrus |
| SM 9 | Left postcentral gyrus |
| SM 2 | Paracentral lobule |
| SM 11 | Right postcentral gyrus |
| SM 27 | Superior parietal lobule |
| SM 54 | Paracentral lobule |
| SM 66 | Precentral gyrus |
| SM 80 | Superior parietal lobule |
| SM 72 | Postcentral gyrus |
| VI 16 | Calcarine gyrus |
| VI 5 | Middle occipital gyrus |
| VI 62 | Middle temporal gyrus |
| VI 15 | Cuneus |
| VI 12 | Right middle occipital gyrus |
| VI 93 | Fusiform gyrus |
| VI 20 | Inferior occipital gyrus |
| VI 8 | Lingual gyrus |
| VI 77 | Middle temporal gyrus |
| CC 68 | Inferior parietal lobule |
| CC 33 | Insula |
| CC 43 | Superior medial frontal gyrus |
| CC 70 | Inferior frontal gyrus |
| CC 61 | Right inferior frontal gyrus |
| CC 55 | Middle frontal gyrus |
| CC 63 | Inferior parietal lobule |
| CC 79 | Left inferior parietal lobule |
| CC 84 | Supplementary motor area |
| CC 96 | Superior frontal gyrus |
| CC 88 | Middle frontal gyrus |
| CC 48 | Hippocampus |
| CC 81 | Left inferior parietal lobule |
| CC 37 | Middle cingulate cortex |
| CC 67 | Inferior frontal gyrus |
| CC 38 | Middle frontal gyrus |
| CC 83 | Hippocampus |
| DM 32 | Precuneus |
| DM 40 | Precuneus |
| DM 23 | Anterior cingulate cortex |
| DM 71 | Posterior cingulate cortex |
| DM 17 | Anterior cingulate cortex |
| DM 51 | Precuneus |
| DM 94 | Posterior cingulate cortex |
| CB 13 | Cerebellum |
| CB 18 | Cerebellum |
| CB 4 | Cerebellum |
| CB 7 | Cerebellum |
