# Supplementary material for: Individualized spatial network predictions using Siamese convolutional neural networks: A resting-state fMRI study of over 11,000 unaffected individuals
Source: PLoS One. 2022 Jan 21;17(1):e0249502. doi: 10.1371/journal.pone.0249502 (PMC8782493; doi:10.1371/journal.pone.0249502)
Supplement: S2 Table — (DOCX) [file pone.0249502.s008.docx]

|  | **Population Number** | **Age (years)** | | | | | | |
| --- | --- | --- | --- | --- | --- | --- | --- | --- |
|  |  | ***Mean*** | ***SD*** | ***Min.*** | ***25%*** | ***50%*** | ***75%*** | ***Max.*** |
| **All** | 7052 (100%) | 62.56 | 7.42 | 46 | 57 | 63 | 68 | 80 |
| **Male** | 3463 (50.9%) | 63.08 | 7.56 | 46 | 57 | 64 | 69 | 79 |
| **Female** | 3589 (49.1%) | 62.07 | 7.26 | 46 | 56 | 62 | 68 | 90 |
