# Supplementary material for: Individualized spatial network predictions using Siamese convolutional neural networks: A resting-state fMRI study of over 11,000 unaffected individuals
Source: PLoS One. 2022 Jan 21;17(1):e0249502. doi: 10.1371/journal.pone.0249502 (PMC8782493; doi:10.1371/journal.pone.0249502)
Supplement: S3 Table — (DOCX) [file pone.0249502.s009.docx]

|  | **Population Number** | **Age (years)** | | | | | | |
| --- | --- | --- | --- | --- | --- | --- | --- | --- |
|  |  | ***Mean*** | ***SD*** | ***Min.*** | ***25%*** | ***50%*** | ***75%*** | ***Max.*** |
| **All** | 2351 (100%) | 62.45 | 7.34 | 45 | 57 | 63 | 68 | 80 |
| **Male** | 1128 (48.0%) | 63.16 | 7.49 | 45 | 57 | 64 | 69 | 80 |
| **Female** | 1223 (52.0%) | 61.80 | 7.14 | 46 | 56 | 62 | 67 | 78 |
