# Supplementary material for: Individualized spatial network predictions using Siamese convolutional neural networks: A resting-state fMRI study of over 11,000 unaffected individuals
Source: PLoS One. 2022 Jan 21;17(1):e0249502. doi: 10.1371/journal.pone.0249502 (PMC8782493; doi:10.1371/journal.pone.0249502)
Supplement: S4 Table — (DOCX) [file pone.0249502.s010.docx]

|  | **Population Number** | **Age (years)** | | | | | | |
| --- | --- | --- | --- | --- | --- | --- | --- | --- |
|  |  | ***Mean*** | ***SD*** | ***Min.*** | ***25%*** | ***50%*** | ***75%*** | ***Max.*** |
| **All** | 2351 (100%) | 62.68 | 7.28 | 47 | 57 | 63 | 68 | 79 |
| **Male** | 1170 (49.8%) | 62.36 | 6.54 | 47 | 57 | 63 | 68 | 79 |
| **Female** | 1181 (50.2%) | 63.01 | 7.55 | 47 | 57 | 64 | 69 | 79 |
