# Supplementary material for: Individualized spatial network predictions using Siamese convolutional neural networks: A resting-state fMRI study of over 11,000 unaffected individuals
Source: PLoS One. 2022 Jan 21;17(1):e0249502. doi: 10.1371/journal.pone.0249502 (PMC8782493; doi:10.1371/journal.pone.0249502)
Supplement: S5 Table — Numbers in parentheses refer to the average accuracy of network pairs with one network of the domain. Numbers in cells are the -"log(p-value)×sign(t)" scores obtained from a two-sided two-sample t-test between mean domain accuracies. The results show that, using Bonferoni correction and a significance level of 0.05, the mean accuracies of different domains are mostly significantly different (Bold cells are significant). Lowest and highest scores are color-coded with blue and red, respectively. The table is symmetric along the main diagonal except for the sign, which reflects the direction of the difference. (B) Cohen’s d associated with Table A. Numbers in cells are the effect size, Cohen’s d, between domain accuracies and are rounded to 3 digits. (DOCX) [file pone.0249502.s011.docx]

| \| **A** \| \| \| \| \| \| \| \| \| --- \| --- \| --- \| --- \| --- \| --- \| --- \| --- \| \|  \| **SC** (79.88) \| **AU** (76.85) \| **SM** (77.75) \| **VI** (78.34) \| **CC** (78.41) \| **DM** (78.52) \| **CB** (77.58) \| \| **SC** \|  \| **64.85** \| **61.47** \| **31.08** \| **37.35** \| **24.51** \| **61.3** \| \| **AU** \| **-64.85** \|  \| **-9.79** \| **-23.47** \| **-26.55** \| **-25.91** \| **-6.31** \| \| **SM** \| **-61.47** \| **9.79** \|  \| **-8.64** \| **-11.93** \| **-11.01** \| 1.11 \| \| **VI** \| **-31.08** \| **23.47** \| **8.64** \|  \| -0.08 \| -0.76 \| **11.33** \| \| **CC** \| **-37.35** \| **26.55** \| **11.93** \| 0.08 \|  \| -0.77 \| **14.68** \| \| **DM** \| **-24.51** \| **25.91** \| **11.01** \| 0.76 \| 0.77 \|  \| **13.74** \| \| **CB** \| **-61.3** \| **6.31** \| -1.11 \| **-11.33** \| **-14.68** \| **-13.74** \|  \|  \| **B** \| \| \| \| \| \| \| \| \| --- \| --- \| --- \| --- \| --- \| --- \| --- \| --- \| \|  \| **SC** \| **AU** \| **SM** \| **VI** \| **CC** \| **DM** \| **CB** \| \| **SC** \|  \| -1.031 \| -0.608 \| -0.41 \| -0.422 \| -0.383 \| -0.793 \| \| **AU** \| 1.031 \|  \| 0.327 \| 0.496 \| 0.511 \| 0.544 \| 0.28 \| \| **SM** \| 0.608 \| -0.327 \|  \| 0.166 \| 0.17 \| 0.203 \| -0.09 \| \| **VI** \| 0.41 \| -0.496 \| -0.166 \|  \| -0. \| 0.033 \| -0.272 \| \| **CC** \| 0.422 \| -0.511 \| -0.17 \| 0. \|  \| 0.034 \| -0.281 \| \| **DM** \| 0.383 \| -0.544 \| -0.203 \| -0.033 \| -0.034 \|  \| -0.316 \| \| **CB** \| 0.793 \| -0.28 \| 0.09 \| 0.272 \| 0.281 \| 0.316 \|  \| |
| --- | --- | --- | --- | --- | --- | --- | --- | --- | --- | --- | --- | --- | --- | --- | --- | --- | --- | --- | --- | --- | --- | --- | --- | --- | --- | --- | --- | --- | --- | --- | --- | --- | --- | --- | --- | --- | --- | --- | --- | --- | --- | --- | --- | --- | --- | --- | --- | --- | --- | --- | --- | --- | --- | --- | --- | --- | --- | --- | --- | --- | --- | --- | --- | --- | --- | --- | --- | --- | --- | --- | --- | --- | --- | --- | --- | --- | --- | --- | --- | --- | --- | --- | --- | --- | --- | --- | --- | --- | --- | --- | --- | --- | --- | --- | --- | --- | --- | --- | --- | --- | --- | --- | --- | --- | --- | --- | --- | --- | --- | --- | --- | --- | --- | --- | --- | --- | --- | --- | --- | --- | --- | --- | --- | --- | --- | --- | --- | --- | --- | --- | --- | --- | --- | --- | --- | --- | --- | --- | --- | --- | --- | --- | --- | --- |
