# Supplementary material for: Individualized spatial network predictions using Siamese convolutional neural networks: A resting-state fMRI study of over 11,000 unaffected individuals
Source: PLoS One. 2022 Jan 21;17(1):e0249502. doi: 10.1371/journal.pone.0249502 (PMC8782493; doi:10.1371/journal.pone.0249502)
Supplement: S6 Table — P-values were computed using the two-sided two-sample t-test to compare male vs. female or same- vs. different-sex cohort. Results show that the mean sensitivity between male and female sub-cohorts are significantly different, especially in CC and CB domains, each of which includes CC-x and SM-x network pairs. Similarly, the p-values of comparing the same- and different cohorts suggest the significance of the mean specificity difference, especially for CC and SM domains. (DOCX) [file pone.0249502.s012.docx]

|  | **Mean Domain Sensitivity (%)** | | | **-log(P-value) (Male-Male vs Female-Female)** | **Mean Domain Specificity (%)** | | | **-log(P-value) (Same- vs Different Sex)** |
| --- | --- | --- | --- | --- | --- | --- | --- | --- |
|  | **Male-Male** | **Female-Female** | **Cohen’s D** |  | **Different Sex** | **Same-Sex** | **Cohen’s D** |  |
| **SC** | 85.94 | 84.55 | 0.19 | 6.121 | 75.3 | 73.67 | 0.22 | 20.465 |
| **AU** | 80.57 | 79.04 | 0.25 | 4.529 | 74.37 | 73.38 | 0.16 | 3.106 |
| **SM** | 81.87 | 80.74 | 0.16 | 7.381 | 75.06 | 73.28 | 0.25 | 35.754 |
| **VI** | 81.8 | 81.31 | 0.07 | 2.059 | 76 | 74.41 | 0.23 | 21.704 |
| **CC** | 82.73 | 81.61 | 0.15 | 11.239 | 75.4 | 73.84 | 0.21 | 51.461 |
| **DM** | 82.68 | 81.65 | 0.14 | 4.915 | 75.72 | 73.99 | 0.24 | 23.947 |
| **CB** | 81.91 | 79.81 | 0.30 | 10.987 | 74.89 | 73.65 | 0.18 | 8.876 |
