# Supplementary material for: Individualized spatial network predictions using Siamese convolutional neural networks: A resting-state fMRI study of over 11,000 unaffected individuals
Source: PLoS One. 2022 Jan 21;17(1):e0249502. doi: 10.1371/journal.pone.0249502 (PMC8782493; doi:10.1371/journal.pone.0249502)
Supplement: S7 Table — P-values were computed using the two-sided two-sample t-test for comparison of young vs. old cohorts. The results depict the mean sensitivity and specificity between the young and old sub-cohorts are significantly different. (DOCX) [file pone.0249502.s013.docx]

|  | **Mean Domain Sensitivity (%)** | | | **-log(P-value) (Young vs. Old)** | **Mean Domain Specificity (%)** | | | **-log(P-value) (Young vs. Old)** |
| --- | --- | --- | --- | --- | --- | --- | --- | --- |
|  | **Age < 52** | **Age > 72** | **Cohen’s D** |  | **Age < 57** | **Age > 69** | **Cohen’s D** |  |
| **SC** | 90.73 | 89.76 | 0.18 | 5.626 | 77.87 | 76.77 | 0.21 | 7.478 |
| **AU** | 86.65 | 85.67 | 0.20 | 3.108 | 77.64 | 75.43 | 0.45 | 8.014 |
| **SM** | 87.21 | 85.42 | 0.31 | 24.651 | 76.58 | 76.20 | 0.07 | 1.754 |
| **VI** | 87.43 | 86.41 | 0.18 | 8.76 | 77.99 | 77.27 | 0.12 | 4.527 |
| **CC** | 88.05 | 86.73 | 0.21 | 22.235 | 77.88 | 76.79 | 0.18 | 17.276 |
| **DM** | 87.80 | 86.29 | 0.26 | 14.063 | 78.21 | 77.12 | 0.19 | 7.198 |
| **CB** | 87.01 | 86.87 | 0.03 | 0.355 | 77.30 | 74.91 | 0.45 | 18.201 |
